# Emergence of the Asian lineage of Zika virus in Angola: an outbreak investigation

**DOI:** 10.1016/S1473-3099(19)30293-2

**Published:** 2019-10

**Authors:** Sarah C Hill, Jocelyne Vasconcelos, Zoraima Neto, Domingos Jandondo, Líbia Zé-Zé, Renato Santana Aguiar, Joilson Xavier, Julien Thézé, Marinela Mirandela, Ana Luísa Micolo Cândido, Filipa Vaz, Cruz dos Santos Sebastião, Chieh-Hsi Wu, Moritz U G Kraemer, Adriana Melo, Bruno L F Schamber-Reis, Girlene S de Azevedo, Amilcar Tanuri, Luiza M Higa, Carina Clemente, Sara Pereira da Silva, Darlan da Silva Candido, Ingra M Claro, Domingos Quibuco, Cristóvão Domingos, Bárbara Pocongo, Alexander G Watts, Kamran Khan, Luiz Carlos Junior Alcantara, Ester C Sabino, Eve Lackritz, Oliver G Pybus, Maria-João Alves, Joana Afonso, Nuno R Faria

**Affiliations:** a Department of Zoology, University of Oxford, Oxford, UK; b Department of Statistics, University of Oxford, Oxford, UK; c Instituto Nacional de Investigação em Saúde, Ministry of Health, Luanda, Angola; d Instituto Nacional de Saúde Doutor Ricardo Jorge, Águas de Moura, Portugal; e University of Lisboa, Faculty of Sciences, BioISI—Biosystems & Integrative Sciences Institute, Lisbon, Portugal; f Departamento de Genética, Instituto de Biologia, Universidade Federal do Rio de Janeiro, Rio de Janeiro, Brazil; g Departamento de Genética, Ecologia e Evolução, Instituto de Ciências Biológicas, Universidade Federal de Minas Gerais, Belo Horizonte, Brazil; h Instituto Superior de Ciências da Saúde, Universidade Agostinho Neto, Luanda, Angola; i Computational Epidemiology Lab, Boston Children's Hospital, Boston, MA, USA; j Harvard Medical School, Boston, MA, USA; k Instituto de Pesquisa Professor Joaquim Amorim Neto, Campina Grande, Brazil; l Department of Human Genetics, Centro Universitário Unifacisa, Campina Grande, Brazil; m Cligest Clinic, Luanda, Angola; n Instituto de Medicina Tropical e Faculdade de Medicina da Universidade de São Paulo, São Paulo, Brazil; o Hospital Pediátrico David Bernardino, Luanda, Angola; p Instituto Nacional de Luta Contra SIDA, Luanda, Angola; q Li Ka Shing Knowledge Institute, St Michael's Hospital, Toronto, ON, Canada; r BlueDot, Toronto, ON, Canada; s Department of Medicine, University of Toronto, Canada; t Laboratório de Flavivirus, IOC-Fundação Oswaldo Cruz/MS, Rio de Janeiro, Brazil; u WHO, Switzerland, Geneva

## Abstract

**Background:**

Zika virus infections and suspected microcephaly cases have been reported in Angola since late 2016, but no data are available about the origins, epidemiology, and diversity of the virus. We aimed to investigate the emergence and circulation of Zika virus in Angola.

**Methods:**

Diagnostic samples collected by the Angolan Ministry of Health as part of routine arboviral surveillance were tested by real-time reverse transcription PCR by the Instituto Nacional de Investigação em Saúde (Ministry of Health, Luanda, Angola). To identify further samples positive for Zika virus and appropriate for genomic sequencing, we also tested samples from a 2017 study of people with HIV in Luanda. Portable sequencing was used to generate Angolan Zika virus genome sequences from three people positive for Zika virus infection by real-time reverse transcription PCR, including one neonate with microcephaly. Genetic and mobility data were analysed to investigate the date of introduction and geographical origin of Zika virus in Angola. Brain CT and MRI, and serological assays were done on a child with microcephaly to confirm microcephaly and assess previous Zika virus infection.

**Findings:**

Serum samples from 54 people with suspected acute Zika virus infection, 76 infants with suspected microcephaly, 24 mothers of infants with suspected microcephaly, 336 patients with suspected dengue virus or chikungunya virus infection, and 349 samples from the HIV study were tested by real-time reverse transcription PCR. Four cases identified between December, 2016, and June, 2017, tested positive for Zika virus. Analyses of viral genomic and human mobility data suggest that Zika virus was probably introduced to Angola from Brazil between July, 2015, and June, 2016. This introduction probably initiated local circulation of Zika virus in Angola that continued until at least June, 2017. The infant with microcephaly in whom CT and MRI were done had brain abnormalities consistent with congenital Zika syndrome and serological evidence for Zika virus infection.

**Interpretation:**

Our analyses show that autochthonous transmission of the Asian lineage of Zika virus has taken place in Africa. Zika virus surveillance and surveillance of associated cases of microcephaly throughout the continent is crucial.

**Funding:**

Royal Society, Wellcome Trust, Global Challenges Research Fund (UK Research and Innovation), Africa Oxford, John Fell Fund, Oxford Martin School, European Research Council, Departamento de Ciência e Tecnologia/Ministério da Saúde/National Council for Scientific and Technological Development, and Ministério da Educação/Coordenação de Aperfeicoamento de Pessoal de Nível Superior.

## Introduction

Zika virus is an RNA virus of the Flavivirus genus that is primarily transmitted by *Aedes* spp mosquitoes. It is classified into two distinct lineages, the African and the Asian genotypes. Serological studies suggest that Zika virus might be widespread across Africa,^1^ but serological assays are difficult to interpret because of extensive cross-reactivity among related flaviviruses.^2^ Before 2007, the virus had been identified in only 14 humans in Africa and Asia,^3^ and infection was thought to cause only mild symptoms, including fever, headache, and rash.^1^ However, since 2013, the Asian genotype of Zika virus has spread to locations in the Pacific and the Americas, resulting in more than 800 000 suspected and confirmed cases of disease.^4^ The discovery that infection with Zika virus during pregnancy can cause severe birth defects and other adverse outcomes^2^ prompted a research response that, to date, has been focused largely on the Pacific and the Americas.

Hundreds of millions of people in sub-Saharan Africa live in areas with competent mosquito vectors and appropriate climatic conditions that render them susceptible to infection with Zika virus.^5^ Despite the potential for widespread circulation, data for transmission of the virus in Africa are scarce. Three African countries (Angola, Cape Verde, and Guinea-Bissau) have reported suspected human cases of Zika virus and clusters of suspected microcephaly cases since 2015.^6^, ^7^, ^8^, ^9^ Only the cases in Angola and Cape Verde are thought to have been caused by the Asian lineage virus.^6^, ^10^, ^11^, ^12^ Understanding these outbreaks is crucial for safeguarding public health in Africa and elsewhere.

**Research in context**

**Evidence before this study**

We searched PubMed with the keywords “Zika” and “Africa” for papers published in any language up to Oct 31, 2018. We also checked available Situation Report publications from WHO up to the same date for evidence of Zika virus or congenital Zika disease in Africa. The African lineage of Zika virus has been detected in Africa since the mid 20th century, yet evidence for spread of the Asian lineage of the virus is scarce. Two countries in Africa (Cape Verde and Angola) have reported cases of Zika virus infection that are thought to have been caused by a newly introduced Asian lineage virus. Sequence data are crucial to confirm and understand the spread of Asian lineage Zika virus in Africa, but these data are currently limited to a single 193 bp fragment of the Zika virus *NS5* gene from Angola. Additionally, although epidemiological data for Zika virus and suspected microcephaly cases in Cape Verde have been reported in detail, data from Angola are extremely scarce.

**Added value of this study**

We sequenced Zika virus genomes from three acutely infected cases, including one baby with confirmed microcephaly. These sequences represent the first three Asian lineage genomes available from Africa. Sequence analysis suggested that Zika virus was probably introduced from Brazil to Angola between July, 2015, and June, 2016, and then circulated for at least 17 months. Our study provides the first genomic confirmation of autochthonous transmission of the Asian lineage of Zika virus within Africa. We also report the second confirmed case of Zika-virus-associated microcephaly in Angola. Our analyses from Angola improve understanding of the extent and clinical effect of Asian lineage Zika virus in Africa.

**Implications of all the available evidence**

The circulation of the Asian lineage of Zika virus in Angola is concerning because of the potential for continued viral spread across Africa. Evidence suggests that Zika virus has circulated and caused microcephaly in Angola and Cape Verde. Detection of additional transmission based on clinical data for suspected cases of microcephaly or clusters of mild illness could be challenging in countries where systems for reporting birth defects are limited and the burden of infectious disease is high. Further spread of the Asian lineage of Zika virus is not likely to be detected unless routine molecular surveillance systems are implemented. Implementation of such a surveillance system is especially important in countries that are linked by high human mobility to areas that have experienced recent outbreaks of Zika virus.

Obtaining accurate surveillance data for Zika virus is challenging because most cases are asymptomatic, symptoms (when present) are mild and non-specific, and infections are frequently misclassified.^2^ In the absence of appropriate surveillance data, phylogenetic analyses of viral genome sequences have proven important in tracing the origins and transmission of outbreaks.^13^, ^14^, ^15^, ^16^, ^17^ The lack of Zika virus genomes from Africa hinders understanding of the re-emergence of the virus on the continent.

In this study, we aimed to investigate the outbreak of Zika virus in Angola. Because surveillance data from Angola are scarce, we considered multiple data streams (surveillance data, genomic data, and human mobility data) that together provide a more complete picture of the outbreak than would be possible had we used any one data type alone. Our goal was to provide the first cohesive insight into the introduction, circulation, and possible public health effects of Zika virus in Angola.

## Methods

### Overview

We did a multi-component investigation into Zika virus and suspected microcephaly cases in Angola. We assessed surveillance data from the Ministry of Health in Angola to identify acute cases of Zika virus infection confirmed by real-time reverse transcription PCR (RT-PCR) between December, 2016, and October, 2018. To identify additional real-time RT-PCR samples positive for Zika virus that are appropriate for sequencing, we also screened samples from 349 people with HIV living in Luanda, Angola, in 2017. We sequenced Zika virus from three samples and did phylogenetic analysis to elucidate the origins and duration of the Angolan outbreak. We analysed human air travel and data on the global incidence of Zika virus infection to support our findings about the geographical source of the introduced strain. Finally, we assessed suspected cases of microcephaly notified to the Ministry of Health in Angola, including an extensive clinical and serological investigation into one case and sequencing of the Zika virus genome from a second (previously identified) case.^10^

### Suveillance for Zika virus in symptomatic, acutely infected cases

The Instituto Nacional de Investigação em Saúde (Ministry of Health, Luanda, Angola) tests cases of suspected acute Zika virus, chikungunya virus, and dengue virus infection. Routine surveillance and diagnostic testing of suspected cases of acute arboviral infections were initiated in late December, 2016. Guidance issued by the Ministry of Health suggests that patients should be suspected of acute Zika virus infection if they present with fever (that cannot be explained by the presence of malaria), conjunctivitis, rash, or arthralgia. Clinical guidance in Angola suggests that patients are suspected of acute infection with dengue virus if they have acute fever (2–7 days' duration), plus at least two of headache, retro-orbital pain, myalgia, arthralgia, exanthema, leucopenia, or haemorrhagic signs. Chikungunya virus infection is suspected if patients have acute fever and unexplained severe arthralgia or arthritis.

### Molecular genetic testing for Zika virus

Serum samples were collected in public health facilities throughout Angola and then transported to the Instituto Nacional de Investigação em Saúde (Luanda, Angola) for centralised real-time RT-PCR (appendix p 4). Viral RNA was extracted using QiaAmp Viral RNA Mini Kits (Qiagen, Germany). Real-time RT-PCR testing for the presence of Zika virus, dengue virus, and chikungunya virus RNA was done with the US Centers for Disease Control (CDC) Trioplex kits^18^ on an Applied Biosystems 7500 Fast machine (Thermofisher Scientific, USA).

### Screening for additional Zika virus-positive samples

To find more samples positive for Zika virus that would be appropriate for Zika virus genomic sequencing, we sought additional samples for real-time RT-PCR screening that were collected between December, 2016, and October, 2017 (ie, when confirmed cases of Zika virus were identified in Angola), and stored at −80°C since sample collection. An appropriate sample set was identified at the Instituto Nacional de Luta Contra SIDA (Luanda, Angola). These samples were collected between April and November, 2017 as part of a separate study investigating antiviral drug resistance among patients with HIV. Participants in this study provided written informed consent for their samples to be used in subsequent studies. No attempt was made to select a demographically representative sample set. We tested these samples for Zika virus with Bio-Manguinhos ZDC real-time RT-PCR kits (Rio de Janeiro, Brazil) on an Applied Biosystems 7500 Fast machine.

### Zika virus genome sequencing

Sequencing of the Zika virus genome of all positive real-time RT-PCR samples was attempted at Instituto Nacional de Investigação em Saúde with an Oxford Nanopore MinION (Oxford, UK) device per previously published protocols.^19^ A consensus sequence was generated with a previously published and validated bioinformatics pipeline (appendix p 5).^19^ Four positive real-time RT-PCR samples were identified in Angola (one sample from the HIV study and three identified from acute cases by the Ministry of Health) but biological material was only available from three. Of these, two samples could be successfully amplified with the attempted whole-genome multiplex PCR.

For genomic sequencing of samples identified in Angola that were positive for Zika virus, we used residual samples without informed consent but with the approval of the Ethical Review Board at the Angolan Ministry of Health. Specifically, residual anonymised clinical diagnostic samples, with no or minimal risk to patients, were provided for research and surveillance purposes within the terms of the Parecer Comité de Ética 06 Julho 2017 (National Ethics Committee, Ministry of Health, Angola).

While investigations in Angola were underway, Zika virus RNA was detected in the urine of a neonate with microcephaly born in Portugal in October, 2017, to a resident of Angola. Details of this case and the appropriate consent and ethical approval have been described elsewhere.^10^ We attempted to sequence the Zika virus genome from that case at the Instituto Nacional de Saúde Doutor Ricardo Jorge, Águas de Moura, Portugal. Therefore, a total of three Zika virus genomes were sequenced during this study.

### Phylogenetic analyses

We did phylogenetic analyses to explore the probable geographical and temporal origins of the Zika virus outbreak in Angola, and the minimal duration of local transmission. We used Muscle to align the three new Zika virus genomic sequences with 390 other viral genomes publicly available from GenBank.^20^ We used PhyML (version 3.1) to estimate maximum likelihood phylogenies^21^ and BEAST (version 1.10.3) to estimate molecular clock phylogenies (appendix p 5).^22^

### Origins of Angolan Zika virus outbreak

An outbreak of Zika virus infection was reported in Cape Verde in 2015–16, from which no genetic sequence data are available.^7^ The absence of genetic data from locations that have reported Zika virus and are connected to Angola by air travel meant that we could not unambiguously infer the geographical origin of the Angolan outbreak with phylogenetic analysis alone. We therefore analysed the global incidence of Zika virus infection and human mobility data to independently investigate the probable source of Zika virus in Angola. Two factors were considered here as contributing to a high risk of exporting the virus to Angola: a high local incidence of Zika virus and high numbers of air passengers travelling to Angola. The monthly number of passengers to Angola from countries reporting Zika virus was estimated on the basis of worldwide ticket sales data from the International Air Transport Association for Jan 1, 2015, to Dec 31, 2017.^23^ The average Zika virus incidence per person per week in each country was estimated from surveillance data for the number of suspected and confirmed cases of Zika virus infection per epidemiological week (appendix p 6).^7^, ^24^

### Surveillance for suspected microcephaly in Angola and association with Zika virus

From Jan 1, 2018, health providers in Angola were required to notify the Angolan Direcção Nacional de Saúde Pública of infants with suspected microcephaly (previously, notification of the Ministry of Health was at clinicians' discretion), and official notification forms were issued. Per guidelines from the Direcção Nacional de Saúde Pública, microcephaly should be suspected in male neonates with head circumference of less than 32 cm and in female neonates with head circumference of less than 31·5 cm, irrespective of gestational age. These measures are equivalent to two SDs below the median head circumference expected for a full-term birth.^25^ Limited coverage of health surveillance across Angola meant that some infants were identified months after birth. In these circumstances, suspected cases of microcephaly were identified on the basis of clinical judgment alone.^26^ We considered all reported cases of suspected microcephaly from whom serum samples were taken, irrespective of whether head circumference was recorded. Thus, all cases identified in Angola that are included in this report are suspected but unconfirmed. Serum samples were also collected from some mothers of suspected cases of microcephaly, at the clinicians' discretion.

Serum samples from suspected cases of microcephaly and mothers of suspected cases were tested for Zika virus, dengue virus, and chikungunya virus by real-time RT-PCR, irrespective of the time between the birth and sample collection, according to the same protocols used for other samples.

Serum samples from eight infants with suspected microcephaly were also tested with the ViDAS ToRC panel (BIOMERIEUX, France) for serological responses to toxoplasmosis, rubella, and cytomegalovirus. The eight infants were randomly chosen from early cases where samples were taken more than 6 days after delivery. Only eight samples were tested due to limited reagent availability.

Serological diagnosis of exposure to Zika virus could not be done in Angola because ELISAs frequently do not accurately discriminate between previous exposure to dengue virus and previous exposure to Zika virus and facilities for conducting more specific tests were unavailable.^27^

### Clinical and serological investigation into a microcephaly case identified in Brazil

We were able to do additional follow-up and confirmation of one suspected case of microcephaly because the girl was identified in Brazil, where a specific serological assay, the plaque reduction neutralisation test, and brain CT and MRI could be done. She was born in Moxico province, Angola, in August, 2017. In November, 2018, she travelled with her mother to the Microcephaly Reference Centre IPESQ (Campina Grande, Brazil), where microcephaly was diagnosed. Before November, 2018, neither the child nor her mother had travelled outside Angola. Plasma samples were taken from both on Nov 30, 2018, and tested for Zika virus and dengue virus infection with the EuroImmun IgG ELISA (London, UK). A plaque reduction neutralisation test was done according to standard protocols (appendix p 4) to quantify neutralising antibodies against Zika virus and dengue virus in both the child and her mother. CT (with a 64-section CT scanner [Philips Brilliance, UK]) and MRI (with a 1.5-T Espree [Siemens Healthcare, Germany]) were done to assess whether the child's neurological damage was consistent with congenital Zika syndrome. Ethical approval for this aspect of the study was granted by the local internal review board at IPESQ (Campina Grande, Brazil; 52888616.4.0000.5693). The mother of the child with microcephaly provided written consent on behalf of herself and her child.

### Role of the funding source

The funders of the study had no role in study design, data collection, data analysis, data interpretation, or writing of the report. The corresponding authors had full access to all the data in the study and had final responsibility for the decision to submit for publication.

## Results

Between December, 2016, and October, 2018, serum samples were collected from 54 patients with suspected Zika virus, who were notified to the Angola Ministry of Health after clinical examination (appendix p 12).

**Figure 1:**
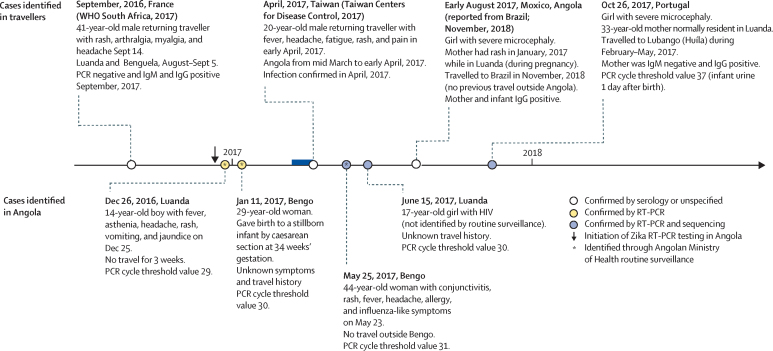
Confirmed Angola-associated cases of Zika virus infection Cases in travellers from Angola identified elsewhere are shown above the line,^10^, ^11^, ^28^ whereas locally identified cases are shown below the line. Dates and locations in bold are the data and place of birth for the girls with microcephaly, and the date and location of sampling for other cases. Cycle threshold values for cases confirmed by real-time RT-PCR are also shown. RT-PCR=reverse transcription PCR.

**Figure 2:**
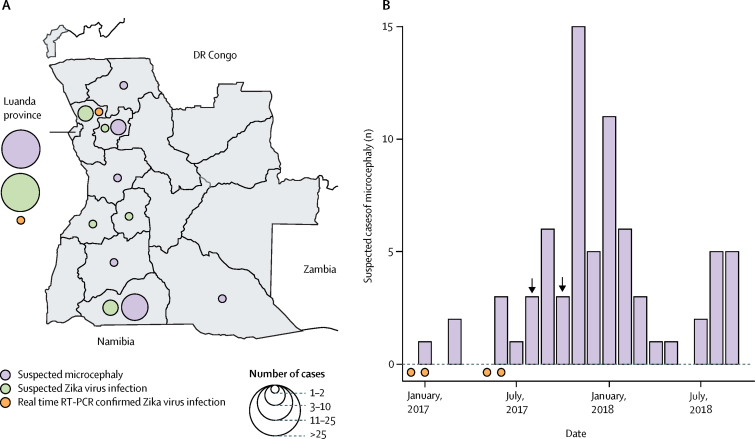
Spatial distribution of suspected cases of acute Zika virus infection and microcephaly (A) and dates of suspected cases of microcephaly (B) in Angola (B) Dates of birth, rather than report dates, are shown, so only infants for whom the date of birth was recorded were included (73 [96%] of 76 cases). Arrows mark the month of birth of the two cases of microcephaly independently identified and confirmed in Brazil (August, 2017), and Portugal (October, 2017).^10^ Orange dots on the horizontal axis show the sampling dates of the four cases of Zika virus infection that were confirmed by real-time RT-PCR in patients who did not have microcephaly. RT-PCR=reverse transcription PCR.

Three cases of Zika virus infection were detected by routine surveillance using real-time RT-PCR (figure 1). The earliest case detected by routine Ministry of Health surveillance was identified in December, 2016, (immediately after real-time RT-PCR surveillance was initiated), and the latest in May, 2017. Data for the time between presence of symptoms and collection of the sample were available for two positive cases (figure 1). Although suspected cases of Zika virus infection were reported across Angola (figure 2A), all confirmed cases of Zika virus infection were residents of, or had travelled to or through, Luanda or the neighbouring province of Bengo (Figure 1, Figure 2).

One additional case of acute Zika virus infection (from June, 2017) was detected by screening an unrelated archive of serum samples from people with HIV using real-time RT-PCR (figure 1; appendix p 12). This person had no history of recent travel outside Luanda province.

No additional positive cases of Zika virus were detected among samples collected during 2018 from the 336 patients with suspected dengue virus or chikungunya virus infections. Of the 51 people with suspected Zika virus infection who tested negative for Zika virus, three tested positive for dengue virus infection, and seven tested positive for chikungunya virus infection. No samples were co-infected with more than one virus.

We did genomic sequencing and phylogenetic analyses of three Zika virus infections acquired in Angola: from one patient notified for suspected Zika virus acute infection, the asymptomatic person detected in the sample from the trial of people with HIV, and a neonate with microcephaly who was previously identified in Portugal.^10^ Figure 1 shows additional information about these three cases, including reported symptoms and travel history (appendix p 13).

Maximum likelihood phylogenies suggested that the three Zika virus strains formed a single, well supported monophyletic clade within the Asian lineage of Zika virus that is circulating in the Americas (bootstrap score 0·96; appendix p 9). Thus, the Zika virus outbreak in Angola probably resulted from a successful introduction of the virus followed by autochthonous transmission within Angola.

**Figure 3:**
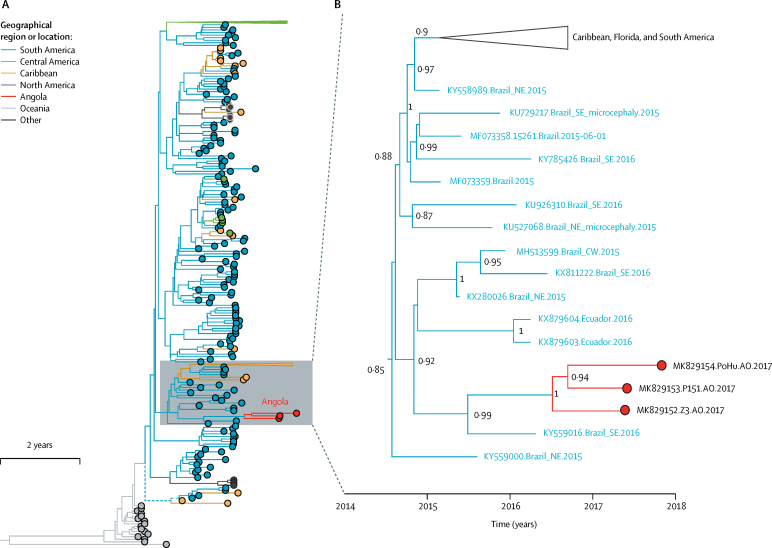
Phylogenetic analysis of the introduction of Zika virus to Angola (A) Maximum clade credibility phylogeny, estimated from complete and near-complete Zika virus genomes with a molecular clock phylogenetic approach. Branch colours indicate the most parsimonious locations of ancestral lineages. Triangular clades represent larger groups of sequences that have been collapsed for visual clarity. (B) Expansion of the clade containing the Angolan Zika virus (red) and closely related sequences from the Americas (blue and yellow). Clade posterior probabilities are shown at well supported nodes.

Our phylogenetic analyses suggest that Zika virus was probably introduced to Angola from Brazil (figure 3), possibly from the southeast of Brazil, where the most closely related virus to the Angola Zika virus clade was sampled (accession number KY559016; posterior probability 0·99). The estimated date of the most recent common ancestor of the Angola Zika virus sequences is June, 2016 (Bayesian 95% credible interval January–October, 2016; figure 3). The date of divergence of the Angolan Zika virus cluster from the most closely related Brazilian Zika virus sequence was July, 2015 (Bayesian 95% credible interval February, 2015–February, 2016; figure 3). Phylogenetic estimates therefore suggest that Zika virus was introduced to Angola from Brazil between July, 2015, and June, 2016. Given that the last positive case was identified in October, 2017 (figure 1)**,** Zika virus therefore probably circulated in Angola for 17–28 months.

**Figure 4:**
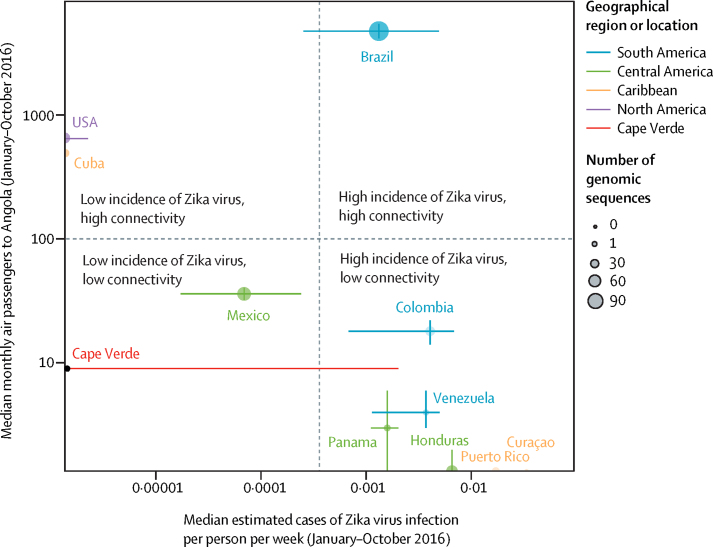
Factors affecting the likelihood of introduction of Asian lineage Zika virus to Angola The 11 countries shown are those with the seven highest median passenger numbers and number of cases of Zika virus per person. Error bars show the IQRs.

Our analysis of global mobility data showed that 80% of all air passengers who travelled to Angola from countries reporting Zika virus infections began their journey in Brazil, whereas only 0·15% began their journey in Cape Verde. Crucially, Brazil was the only country that was highly connected to Angola via air travel and had a high frequency of cases of Zika virus infection (figure 4). Furthermore, the climatic suitability for *Aedes* spp mosquito-borne viral transmission is more synchronous between Angola and Brazil than between Angola and Cape Verde (appendix p 10). Thus, genomic, epidemiological, mobility, and climatic data support the hypothesis that Zika virus was introduced directly to Angola from Brazil.

Serum samples were collected from 76 infants with suspected microcephaly between Jan 1, 2017, and Nov 31, 2018 (median time of sample collection 24 days after birth [IQR 4–85; range 0–315 days]; figure 2; appendix p 7). The number of neonates with suspected microcephaly peaked around November, 2017, and subsequently declined (figure 2B). This peak occurred several months after PCR-confirmed cases of acute Zika virus infection were detected in Angola (figure 2B). Head circumference was not recorded for any of the 14 suspected cases of microcephaly that were detected in 2017, but was recorded for 53 (85%) of the 62 suspected cases reported in 2018. All infants with suspected microcephaly for whom head circumference was measured had head circumferences that were at least two SDs below expected by age according to WHO's child growth standards (appendix p 11), regardless of time of identification after birth. Additional clinical assessments were not typically reported. Serum samples were also collected from 24 mothers.

No samples from infants with suspected microcephaly or their mothers were positive for Zika virus infection as measured by real-time RT-PCR. Conversely, none of the eight infants in whom serological testing was done showed evidence of recent infection with other ToRC pathogens. Thus, the cause of microcephaly was uncertain.

The girl with microcephaly who was born in Angola but presented in Brazil on whom we did extensive investigations had a head circumference at birth of 29 cm (Z score −3·6) according to her mother. Clinical examination suggested severe and disproportionate microcephaly. The 21-year-old mother was a long-term resident of Moxico province, but travelled to Luanda during the second and third months of pregnancy and developed a rash during this visit (week 10 of pregnancy; January, 2017). Diagnostic surveillance data showed that Zika virus was present in Luanda and Bengo province when she had this rash (figure 1). The girl with microcephaly was born in August, 2017, coincident with the increase in neonates with suspected microcephaly reported in Angola (figure 2).

**Figure 5:**
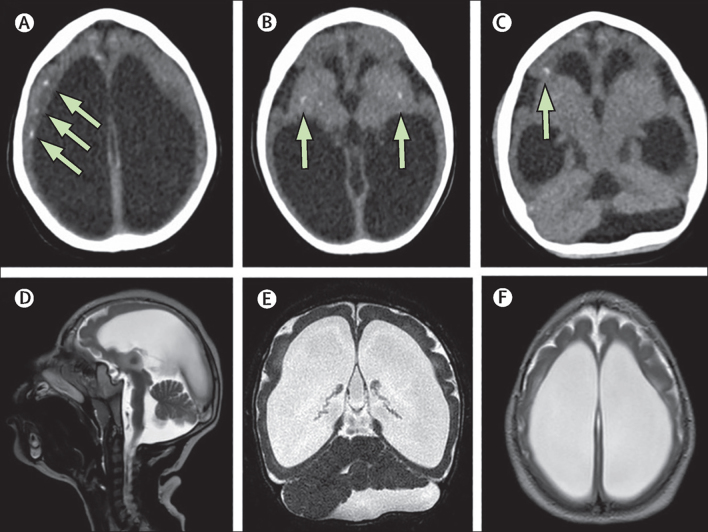
Brain CT and MRI scans of an Angolan child with microcephaly (A) Compensatory ventriculomegaly and calcification areas in the subcortical region are shown by green arrows. In (B) and (C), calcification in the basal ganglia is shown by the green arrows. (D) Brainstem hypoplasia. (E) Dysgenesis of the cerebellum. (F) Pachygyria.

CT and MRI, which were done when the child was age 15 months, confirmed microcephaly via reduced cerebral volume, and showed abnormalities consistent with congenital Zika syndrome that had been noted in Brazil and elsewhere—eg, calcification areas, ventriculomegaly, brainstem hypoplasia, dysgenesis of the cerebellum, and pachygyria (figure 5).^29^ At age 15 months, the child had a head circumference of 37 cm (Z score −6·3). She was IgG negative for both dengue virus and Zika virus by ELISA, but weakly positive for Zika virus neutralising antibodies according to the specific plaque reduction neutralisation test (titre=40). Her mother was strongly IgG positive for Zika virus by ELISA, and had a weak dengue virus IgG response. A specific plaque reduction neutralisation test confirmed the strong neutralising antibody response in the mother (titre=1280). These findings strongly suggest that the mother had been previously infected with Zika virus. Both the mother and child had negative dengue virus neutralising antibody responses by specific plaque neutralisation test assay (titre ≤10). The long-term outcomes for this child included delayed neurological and development status (on the Bayley Scales of Infant and Toddler Development) and epileptic spasms—findings common in cases of congenital Zika syndrome.

## Discussion

In this multi-component outbreak investigation, we characterised the first known outbreak of Asian lineage Zika virus in continental Africa. Although surveillance, genetic, and clinical data were individually scarce, together these independent lines of evidence provide a cohesive picture of the outbreak of Zika virus infection in Angola. We report data for five cases of Zika virus infection between December, 2016, and October, 2017, and confirmed by real-time RT-PCR. The three Zika virus genomes that we sequenced represent the first Asian lineage genomes reported from Africa, and the first genome isolated from a case with Zika-virus-associated microcephaly in Africa. Our phylogenetic analyses showed that Zika virus was probably introduced to Angola from Brazil and then circulated for 17–28 months. Finally, our serological investigations in one mother–child pair supported the view that the Zika virus outbreak caused microcephaly in Angola.

Phylogenetic analysis showed that the three Angolan Zika virus genomes formed a single clade with a common ancestor in June, 2016. These findings suggest either a single successful introduction that initiated local Zika virus transmission in Angola that continued until at least June, 2017, or recurrent but later introduction to Angola of viruses belonging to a specific lineage present in Brazil. The latter explanation is much less likely, because it is improbable that three or more independent introductions of Zika virus to Angola would belong to only one of the many different lineages that circulated contemporaneously in Brazil.^13^ Thus, Zika virus could have circulated in Angola for at least 3 months before a case was first detected in a returning traveller,^11^ and for at least 6 months before a local case was detected. Similar or longer periods of cryptic Zika virus transmission have been reported in the Americas^13^ and attributed partly to difficulties in identifying clinical cases when infections are asymp-tomatic or mildly symptomatic.^15^

Zika virus probably circulated in Angola for 17–28 months—sustained transmission that implies that the outbreak was substantially larger than the small number of cases detected by surveillance. Low case detection has been reported previously (eg, in Cape Verde, where less than 3% of the estimated number of infected individuals were suspected to have Zika virus infection).^7^ Retrospective screening of stored samples could help to establish the magnitude and duration of undetected Zika virus transmission in Angola since 2015.

Zika virus was probably introduced to Angola from Brazil, but we cannot rule out the possibility that it could have spread to Angola from affected locations where genomic data are unavailable. However, data for human flight mobility and the global incidence of Zika virus support our phylogenetic conclusion that Brazil is the most likely origin of the Angolan outbreak. Transmission of mosquito-borne viruses between these two countries was previously shown by the spread of chikungunya virus from Angola to Brazil in 2014.^30^ Notably, both African countries with confirmed Asian-lineage Zika virus (ie, Angola and Cape Verde) have regular air connectivity with Brazil,^5^ which likely reflects the close historical, cultural, and linguistic links between these countries. Of all African countries, Angola received the largest number of travellers from Zika-virus-affected countries in the Americas.^5^ The introduction of Zika virus to Angola therefore underscores the need to coordinate viral surveillance strategies across countries that share high human interconnectivity and similar vector-borne transmission potential, irrespective of their distance apart.

The Asian lineage of Zika virus that circulated in Angola caused microcephaly in Brazil and elsewhere.^31^ In our study, none of the 76 infants with suspected microcephaly whom we tested were positive for Zika virus infection by real-time RT-PCR. However, this finding does not prove that all these infants did not have previous Zika virus infection. Zika viraemia declines to undetectable levels in blood within 11 days of symptom onset in adults.^32^ The infant samples tested here were collected a median of 24 days after birth, and infection could have occurred early during pregnancy. The occurrence of several, large dengue virus outbreaks in Angola^33^, ^34^ meant commercial ELISAs could not be used to serologically diagnose Zika virus, because these assays frequently fail to accurately discriminate between previous exposure to dengue virus and to Zika virus.^27^ The cause and clinical severity of the suspected cases of microcephaly identified by routine surveillance by the Angola Ministry of Health that we report here remain unclear.

To date, there have been only two confirmed reports of microcephaly in which the mother of the infant lived in Angola: one infant who was identified clinically in this study, and one who was identified by Sassetti and colleagues^10^ for whom we sequenced the infecting Zika virus genome. Both infants had brain abnormalities consistent with congenital Zika syndrome. The mother of the child identified in our study had a strong Zika-virus-positive response on the specific plaque neutralisation test assay, by contrast with her child, who had only a weakly positive response. This result is consistent with intrauterine Zika virus exposure because infants typically lose maternally acquired IgG antibodies during the 6–12 months after birth,^35^ and the child was 15 months old when tested. Data for the two confirmed cases therefore strongly suggest that Zika virus has caused microcephaly in Angola. Both infants were born at approximately the same time as the rise in suspected microcephaly cases identified independently by the Angolan Ministry of Health.

We present several complementary lines of evidence that together form a cohesive insight into the origins and effects of Zika virus in Angola. Despite the overall concordance of these different data streams, several aspects of the study have additional limitations that should be considered. First, the proportion of diagnostic samples that were positive for Zika virus infection by real-time RT-PCR in Angola is probably an underestimate of the true numbers of infections. Suboptimum sample transport and lack of information about the date of onset of symptoms mean that samples could have degraded before testing or been collected after viraemia was no longer detectable.^32^ Second, the spatiotemporal distributions of notified suspected cases of microcephaly and Zika virus were very difficult to interpret with confidence because the total number of reported cases was low, the consistency of case detection and reporting were probably highly variable, and the intensity of surveillance efforts has changed over time. Gestational age was not recorded for any suspected cases of microcephaly, and head circumference was not reported for some notified cases, and thus the severity of microcephaly was difficult to establish. The absence of serological testing for Zika virus and other STORCH pathogens (ie, syphilis, *Toxoplasma gondii*, rubella, cytomegalovirus, herpes simplex, and others) in infants with suspected microcephaly makes the cause of these cases difficult to establish. Widespread, routine screening of pregnant women for STORCH pathogen infections and follow-up of children born with brain abnormalities would improve understanding of the extent and causes of birth defects in Africa.

## Data sharing

Sequence alignments, XMLs, and tree files are all publicly available on GitHub. Zika virus genome sequences are available on GenBank with accession numbers MK829152-MK829154.
