# Supplementary material for: Emergence of the Asian lineage of Zika virus in Angola: an outbreak investigation
Source: Lancet Infect Dis. 2019 Oct;19(10):1138–47. doi: 10.1016/S1473-3099(19)30293-2 (PMC6892302; doi:10.1016/S1473-3099(19)30293-2)
Supplement: Supplementary appendix [file mmc1.pdf]

THE LANCET Infectious Diseases

Supplementary webappendix

This webappendix formed part of the original submission and has been peer reviewed. We post it as supplied by the authors.

Supplement to: Hill SC, Vasconcelos J, Neto Z, et al. Emergence of the Asian lineage of Zika virus in Angola: an outbreak investigation. *Lancet Infect Dis* 2019; **19:** 1138–47.

Supplementary Appendix: Emergence of the Zika virus Asian lineage in Angola

Sarah C. Hill, D.Phil. [1], Jocelyne Vasconcelos, M.Sc. [2], Zoraima Neto, Ph.D. [2], Domingos Jandondo, B.Sc. [2], Líbia Zé-Zé, Ph.D. [3,4], Prof Renato Santana Aguiar, Ph.D. [5,6], Joilson Xavier, B.Sc. [6], Julien Thézé , Ph.D. [1], Marinela Mirandela, B.Sc. [2], Ana Luísa Micolo Cândido, B.Sc. [2], Filipa Vaz, M.Sc. [2], Cruz dos Santos Sebastião, M.Sc. [2,7], Chieh-Hsi Wu, Ph.D. [8], Moritz U.G. Kraemer, D.Phil. [1,9,10], Prof Adriana Melo, Ph.D.[11], Bruno L.F. Schamber-Reis, Ph.D. [12], Girlene S. de Azevedo, M.Sc. [11], Prof Amilcar Tanuri M.D., Ph.D. [5], Luiza M. Higa, Ph.D. [5], Carina Clemente M.Sc. [13], Sara Pereira da Silva, M.Sc. [13], Darlan da Silva Candido, M.Sc. [1], Ingra M. Claro, B.Sc. [14], Domingos Quibuco, Nurse [15], Cristóvão Domingos, B.Sc., [16], Bárbara Pocongo, M.Sc. [16], Alexander G. Watts, Ph.D. [17,18], Prof Kamran Khan, M.D. [17,18,19], Prof Luiz Carlos Junior Alcantara, Ph.D. [6,20], Prof Ester C. Sabino, Ph.D. [14], Eve Lackritz, M.D. [21], Prof Oliver G. Pybus, D.Phil [1], Maria-João Alves, Ph.D. [3], Joana Afonso, Ph.D. [2*], Nuno R. Faria, Ph.D. [1*]

1) Department of Zoology, University of Oxford, U.K.
2) Instituto Nacional de Investigação em Saúde, Ministry of Health, Luanda, Angola.
3) Instituto Nacional de Saúde Doutor Ricardo Jorge, Águas de Moura, Portugal.
4) University of Lisboa, Faculty of Sciences, BioISI - Biosystems & Integrative Sciences Institute, Lisboa, Portugal.
5) Departamento de Genética, Instituto de Biologia, Universidade Federal do Rio de Janeiro, Brazil.
6) Departamento de Genética, Ecologia e Evolução, Instituto de Ciências Biológicas, Universidade Federal de Minas Gerais, Brazil.
7) Instituto Superior de Ciências da Saúde, Universidade Agostinho Neto, Luanda, Angola.
8) Department of Statistics, University of Oxford, UK
9) Computational Epidemiology Lab, Boston Children's Hospital, Boston, USA
10) Harvard Medical School, Boston, USA
11) Instituto de Pesquisa Professor Joaquim Amorim Neto (IPESQ), Campina Grande, Brazil.

12) Department of Human Genetics, Centro Universitário Unifacisa, Campina Grande, Brazil.
13) Cligest Clinic, Luanda, Angola.
14) Instituto de Medicina Tropical e Faculdade de Medicina da Universidade de São Paulo, São Paulo, Brazil.
15) Hospital Pediátrico David Bernardino, Luanda, Angola
16) Instituto Nacional de Luta Contra SIDA, Luanda, Angola
17) Li Ka Shing Knowledge Institute, St. Michael's Hospital, Toronto, Canada
18) BlueDot, Toronto, Canada.
19) Department of Medicine, University of Toronto, Canada
20) Laboratório de Flavivirus, IOC-Fundação Oswaldo Cruz/MS, Rio de Janeiro, Brazil.
21) World Health Organization, Switzerland, Geneva

* Corresponding author: jmafonso.7@gmail.com and nuno.faria@zoo.ox.ac.uk

Contents:

Page 4: *Supplementary Materials and Methods.*

Page 7: *Supplementary Figure 1*. Number of days between birth and sample collected for suspected microcephaly cases.

Page 8: *Supplementary Figure 2*. Root to tip regression of sequence sampling date and genetic divergence and maximum likelihood phylogeny.

Page 9: *Supplementary Figure 3.* Maximum likelihood phylogeny of ZIKV

Page 10: *Supplementary Figure 4.* Seasonal climatic suitability for *Aedes aegypti*.

Page 11: *Supplementary Figure 5.* Cephalic perimeter of sampled suspected microcephaly cases by age and sex.

Page 12: *Supplementary Table 1*. Summary of sample testing and positive samples detected.

Page 13: *Supplementary Table 2*. Sequencing statistics for Angolan ZIKV genomes.

Page 14: *References.*

Supplementary Materials and Methods

Collection and transport of samples

Sample storage conditions between sample collection at public health facilities and sample receipt at Instituto Nacional de Investigação em Saúde (INIS) were not recorded and would likely have been variable for different samples. Samples were often transported at room temperature because of limited availability of frozen cool blocks in many public health facilities. Samples were typically received at INIS 0-1 days after sample collection, but in rare cases were received several weeks after collection (median and interquartile range data in **Table S1**).

Where possible, RNA was extracted from samples and real-time reverse transcription PCR (rRT-PCR) performed immediately upon sample receipt. When this was not possible, samples were frozen at -20C for up to ~24 hours prior to RNA extraction, and/or extracted RNA was frozen at -80C prior to rRT-PCR.

Although requested on paper notification forms, the date of onset of symptoms and clinical descriptions of symptoms were not available for most suspected acute ZIKV, CHIKV or DENV cases. It is therefore not possible to formally assess either clinical adherence to case notification guidelines, or the time between onset of symptoms and sample collection, using these data.

ZIKV diagnostic testing

Samples from the Instituto Nacional de Luta Contra Sida were pooled in triplicate prior to RNA extraction and rRT-PCR, followed by individual testing of samples if a pool was positive. Testing of these, and other samples, is described in the **Main Text**.

PRNT on microcephalic infant and mother

Plaque reduction neutralisation tests (PRNT) were performed to quantitate neutralising antibodies against ZIKV and DENV2. Briefly, plasma samples were heat-inactivated at 58°C. Two-fold dilutions of heat-inactivated plasma (ranging from 1:5 to 1:2560) were incubated with 100 plaque forming units (PFU) of ZIKV (strain MR766) or DENV2 (strain 16681) for 1 hour at 37°C. The virus-plasma mixture was inoculated onto confluent monolayers of VERO cells for ZIKV PRNT or PS cells for DENV2 PRNT. In addition, virus-only controls were included to determine the infectivity of the challenge virus. After 1 hour, inoculum was removed, and cells were overlaid with semisolid medium (1.25% carboxymethylcellulose in alpha-MEM supplemented with 1% fetal bovine serum) and then further incubated at 37°C for 5 days. Cells were fixed with 4% formaldehyde solution and stained with crystal violet dye solution for plaque visualisation. The plaque reduction neutralisation titre was defined as the highest serum dilution that results in 90% reduction (PRNT90) of infectivity when compared with the challenge virus.

ZIKV sequencing and consensus sequence generation

Sequencing of coding regions of the ZIKV genome was attempted using an Oxford Nanopore Technology (ONT) MinION device following previously published methods.[1] Briefly, cDNA was generated from viral RNA using random hexamers Protoscript II First Stand cDNA Synthesis kit or Superscript IV First Strand Synthesis System. Multiplex PCR with 42 cycles was used to generate overlapping amplicons that spanned the whole coding region of the ZIKV genome, according to previously published thermocycling conditions.[1] PCR products were purified using 1x Ampure XP beads. The concentration of DNA was quantified using a Qubit Fluorometer, and samples were standardised by concentration. Library preparation was performed using 200-350 ng total mass of PCR product as input. Library preparation was performed using the ONT SQK-LSK108 ligation sequencing kit and NBD103 Native Barcoding Kit according to the manufacturer's instructions but with the changes detailed in [1] and https://www.protocols.io/view/one-pot-ligation-protocol-for-oxford-nanopore-libr-k9acz2e. The library was loaded onto FLO-MIN106 flow cells, and sequencing conducted without basecalling for 12-48 hours using MinKNOW 2 software. Negative controls were also sequenced.

Raw sequencing data were processed according to established pipelines.[1] Raw reads were basecalled using Albacore (Oxford Nanopore Technologies), demultiplexed and adaptor-trimmed using Porechop, and mapped to a reference genome (GenBank accession number KJ776791) using bwa v 0.7.16a-r1181. Nanopolish software was used to identify variation from this reference genome. A consensus was generated for all genomic sites where coverage was at least >20X. We have shown previously that 20x coverage of Nanopore MinION data is sufficient to generate ZIKV consensus genomes that are identical to those produced using the Illumina MiSeq platform, regardless of the MinION chemistry version used (R7.3, R9, R9.4). [2]

Phylogenetic analysis

The maximum likelihood tree was estimated using PhyML v3.1[3] under a general time reversible nucleotide substitution model, with gamma-distributed among-site rate variation and a proportion of invariant sites (GTR + G + I). Appropriate temporal signal for estimation of molecular clock phylogenies was assessed using TempEst [4] (**Figure S2**). Phylogenies calibrated in time units were estimated under a relaxed clock model and a codon-partition (SRD06) nucleotide substitution model [5] for ZIKV using the MCMC approach implemented in BEAST v1.10.3.[6] Three independent MCMC chains of 250 million steps were computed, sampled every 25,000 steps. The first 10% of each run was discarded as burn-in, convergence of the runs was checked using Tracer 1.7.1,[7] and maximum clade credibility trees were constructed using TreeAnnotator.[6] Alignments, XMLs and tree files are available at GitHub (https://github.com/arbospread/Zika_Angola).

Estimation of the origins of Angolan ZIKV

To further investigate the geographic origin of ZIKV in Angola, countries that could have exported the ZIKV lineage to Angola were identified using multiple data sources. Specifically, two factors were considered here as resulting in a high risk of introducing ZIKV to Angola: (i) countries with a high local incidence of ZIKV, and (ii), countries with high number of passengers travelling by air into Angola. Countries in which ZIKV Asian lineage confirmed cases were detected during 2015-2016 were identified from WHO reports.[8] For each of these countries, the number of passengers to Angola was estimated by analysing worldwide air ticket sales data from the International Air Transport Association (IATA) during 2015-2017.[9] These data included the full itineraries of travellers: their initial airport of embarkation, their final destination airport and, where applicable, connecting airports, but did not detail incomplete trips (e.g., due to missed flights).

Records of the number of suspected or confirmed cases of ZIKV from each country in the Americas that has reported ZIKV cases during the likely period of ZIKV introduction to Angola were used as a measure of local ZIKV incidence. Data from countries in Asia were not included as we show later that the Angolan ZIKV strain clearly belongs to the lineage of the Asian ZIKV genotype circulating in the Americas (**Figure 3**). Surveillance data for the Americas included suspected and confirmed cases of ZIKV per epidemiological week reported to PAHO.[10] Equivalent data for Cabo Verde, the only other country in Africa reporting the ZIKV Asian lineage, were taken from publicly available official reports of the Surveillance and Outbreak Response Unit of the Ministry of Health of Cabo Verde (SVIER-MS).[11,12] Only suspected and confirmed cases that occurred around the time of the most recent common ancestor of ZIKV strains in Angola were considered (as estimated using our molecular clock analysis). To provide a crude estimate of infection risk per person, here we scale the number of reported cases by the population size in each country in 2015.[13]

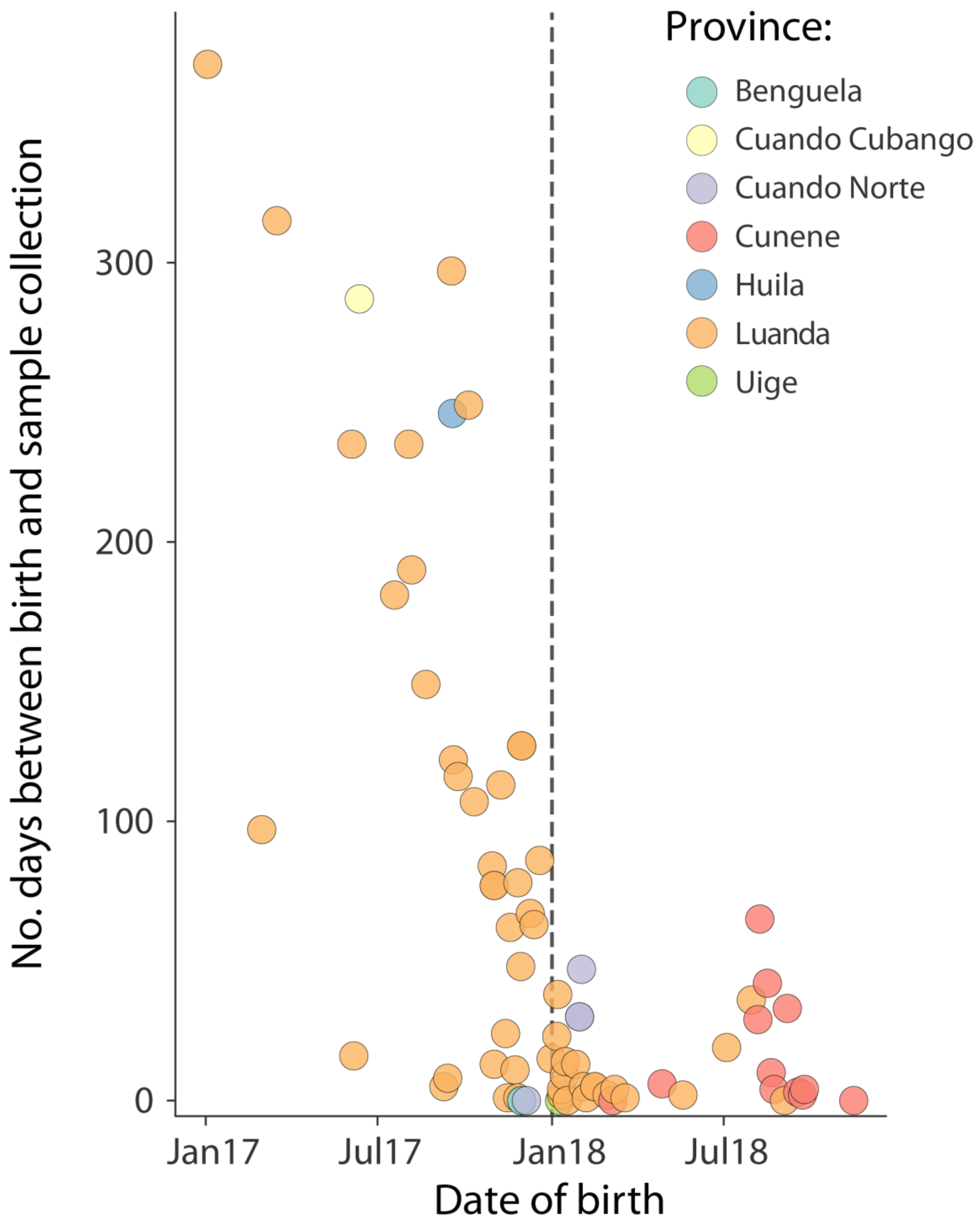

Supplementary Figure 1. Number of days between birth and sample collected for suspected microcephaly cases. Colours indicate province of sampling. Dashed vertical line indicates implementation of requested case reporting of babies with suspected microcephaly in Angola. Most cases identified prior to this time were identified retrospectively.

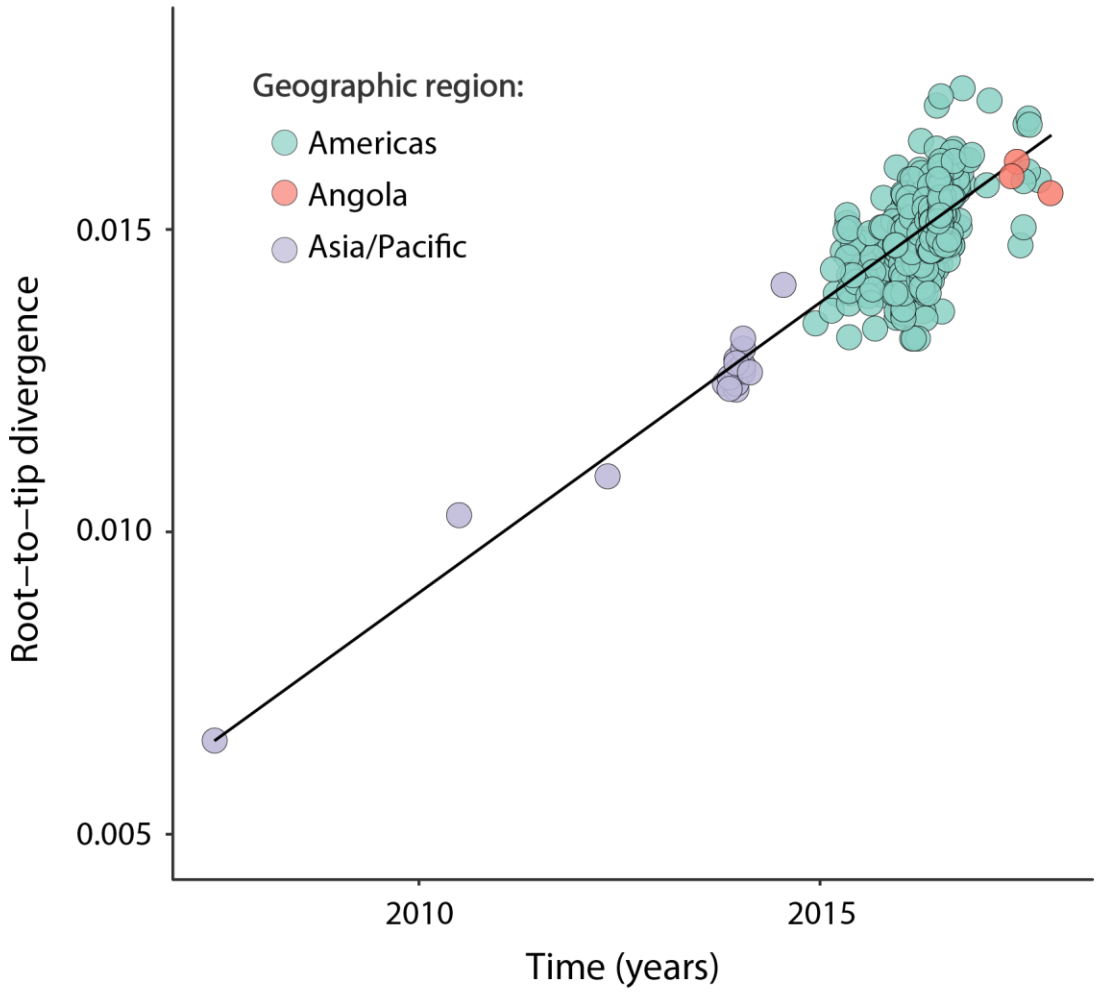

Supplementary Figure 2. Root to tip regression of sequence sampling date and genetic divergence and maximum likelihood phylogeny. Sequences are coloured by sampling location. Here, the geographic region 'Americas' includes ZIKV genomes from the Caribbean and ZIKV genomes from lineages that were likely exported from the Americas (for example, in returning travellers). 'Asia/Pacific' refers to all other Asian lineage ZIKV genomes sampled in 2014 or earlier, and includes genomes from French Polynesia, Micronesia, Cambodia, Thailand and the Philippines.

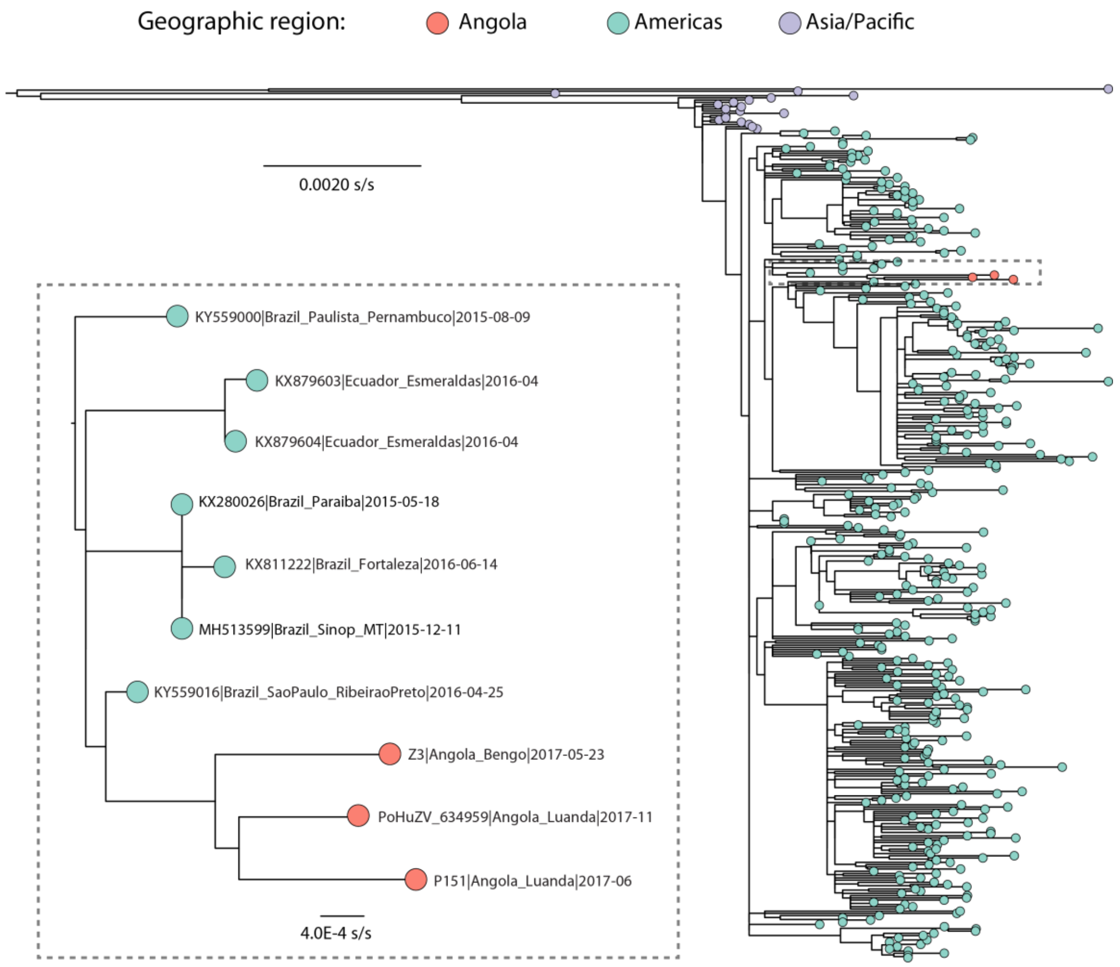

Supplementary Figure 3. Maximum likelihood phylogeny of ZIKV. The tree was estimated using 393 complete and partial ZIKV genomes (see Methods for details). The clade containing the Angola sequences is expanded on the bottom left. Sequences are coloured by sampling location (as in Supplementary Figure 2).

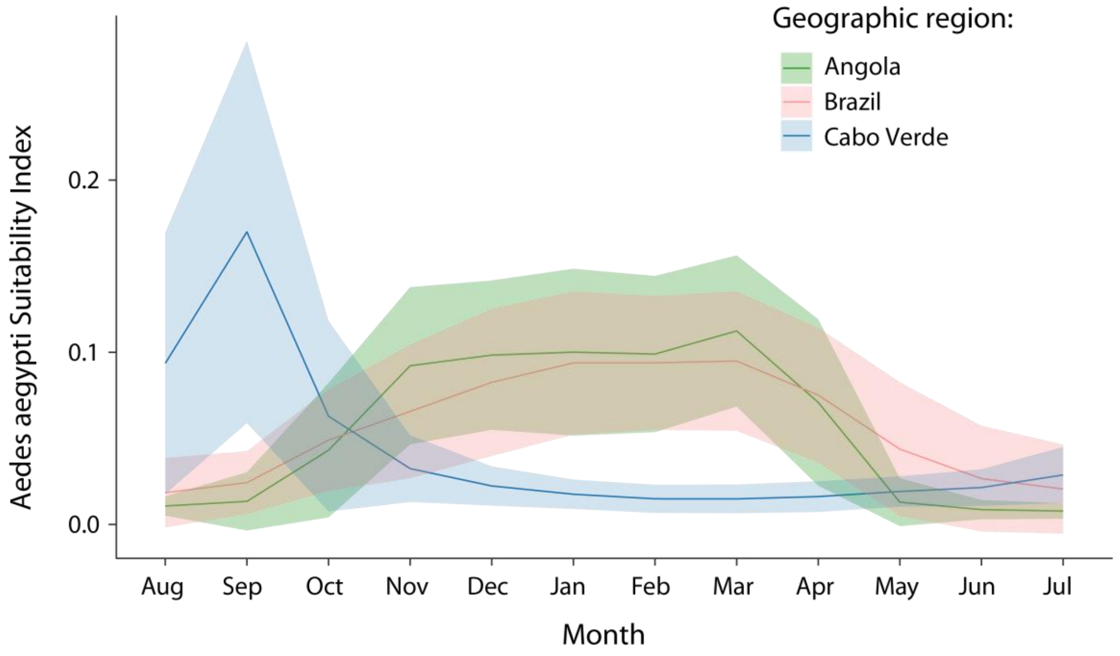

Supplementary Figure 4. Seasonal climatic suitability for *Aedes aegypti*. Solid lines represent the mean climatic suitability in that month and shaded areas show 68% confidence intervals. Data were obtained from [14]. Mean suitability per country was calculated using the R *raster* package (https://cran.r-project.org/web/packages/raster/index.html)

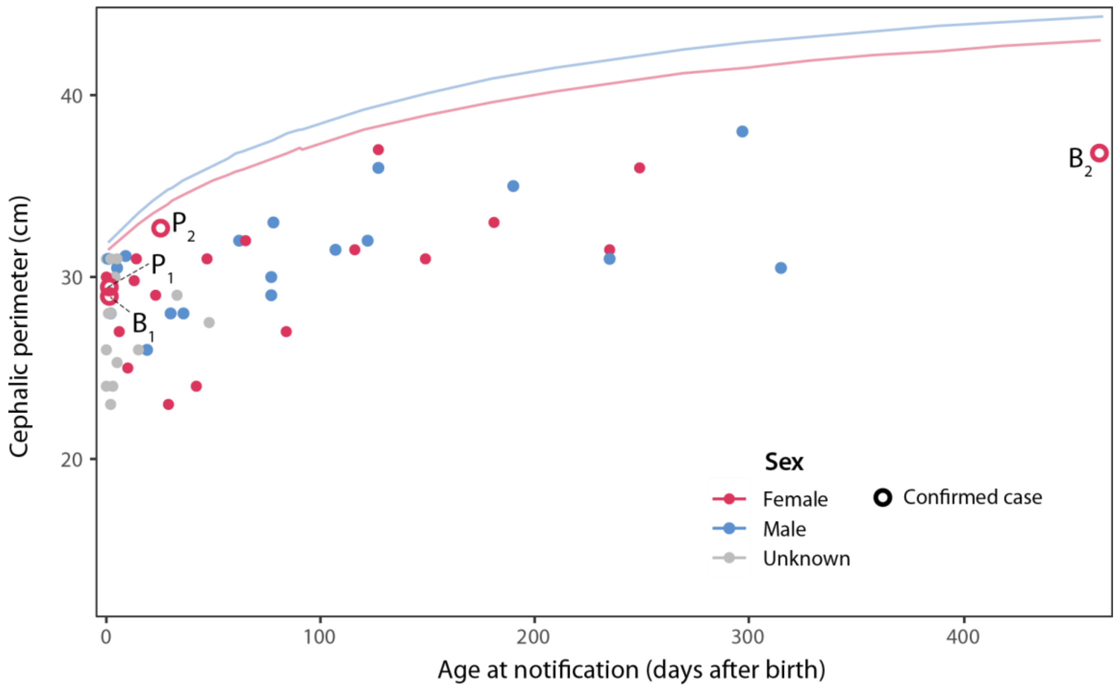

Supplementary Figure 5. Cephalic perimeter of sampled suspected microcephaly cases by age and sex. Colours indicate male (blue), female (red) and unknown sex (grey) cases, respectively. Solid circles represent suspected cases of microcephaly notified in Angola. Only suspected cases for which cephalic perimeter was recorded are shown. Open circles represent two ZIKV associated confirmed microcephaly cases from Angola, who were measured at different ages. These cases were identified in Brazil (B_1 and B_2) and Portugal (P_1 and P_2 [15]), and are included here for comparison (see Manuscript text for additional details). Lines represent 2 standard deviations below the WHO Child Growth Standards of expected head circumference-for-age. We stress that the -2SD standard does not represent an official WHO definition of microcephaly and is included here for reference only.

Supplementary Table 1. Summary of sample testing and positive samples detected.

Sample set	Date range	Number of samples[1]	Assay method	Number of positive samples (percentage)	Median days between sample collection and receipt by INIS (IQR)
Symptomatic patients with suspected acute ZIKV infection	Late Dec 16 – Nov 18	54	CDC Trioplex rRT-PCR (ZIKV/CHIKV/DENV)	3/54 (6 %)	1 (3)
Symptomatic patients with suspected acute DENV infection	Jan 18 – early Nov 18	330	CDC Trioplex rRT-PCR (ZIKV/CHIKV/DENV)	0 /330(0 %)	4 (5)[2]
Symptomatic patients with suspected acute CHIKV infection	Sept-Oct 18	6	CDC Trioplex rRT-PCR (ZIKV/CHIKV/DENV)	0/6 (0 %)	3 (0)
Infants with suspected microcephaly	Jan 17 – Oct 18	76	CDC Trioplex rRT-PCR (ZIKV/CHIKV/DENV)	0/76 (0 %)	1 (3)
Mothers of infants with suspected microcephaly	Jan 17 – Oct 18	24	CDC Trioplex rRT-PCR (ZIKV/CHIKV/DENV)	0/24 (0 %)	2 (4)
Samples from HIV+ patients	April – Nov 17	349	ZDC rRT-PCR (ZIKV only)	1/349 (0.3 %)	Not applicable[3]

Notes:
1) One sample was tested per unique individual. All samples were serum samples.
2) Median and IQR is longer for this group, because 43 samples were stored at -20C at the site of sample collection for 3 months, prior to shipment to INIS.
3) These samples were collected and stored at -80C from collection in 2017 to testing in 2018 at the Instituto Nacional de Luta Contra SIDA.

Supplementary Table 2. Sequencing statistics for Angolan ZIKV genomes. Note that a short 193bp fragment of the ZIKV NS5 gene from one patient (a microcephalic neonate born in Portugal) was previously reported (GenBank accession number MG742364).[15] As expected, this fragment exactly matches the relevant section of the larger genome sequence reported here, confirmed by a synonymous mutation observed in both sequences that is not seen in any other sampled ZIKV Asian lineage or African lineage genomes. The same patient sample that was used to obtain the short 193bp fragment (PoHuZV/634959) was used again in this study for genomic sequencing.

The three Angolan ZIKV sequences differ from each other at only 34 nucleotide sites. Three of these variable sites cause amino acid changes: Y135H (in sample Z3), Y3038H and D3344E) (both in sample PoHuZV_634939, isolated from a microcephaly patient [15]). Neither of the two amino acid changes observed in strain PoHuZV_634939 are seen in either of the two most closely related ZIKV genomes obtained from infants born with microcephaly in Brazil (GenBank accession numbers KU729217, KU527068).

**Sample (GenBank accession number)**	**Genomic coverage (%)**	**Mapped reads**	**Average depth**	**Bases covered >10x**	**Bases covered >25x**
Z3 (MK829152)	64·3	215916	3051	8910	8287
P151 (MK829153)	80·8	17162	714	9236	8924
PoHuZV634959 (MK829154)	88·6	530192	3961	10249	10193

References

1 Quick J, Grubaugh ND, Pullan ST, *et al.* Multiplex PCR method for MinION and Illumina sequencing of Zika and other virus genomes directly from clinical samples. *Nat Protoc* 2017; **12**: 1261–76.

2 Faria NR, Quick J, Claro IM, *et al.* Establishment and cryptic transmission of Zika virus in Brazil and the Americas. *Nature* 2017; **546**: 406–10.

3 Guindon S, Dufayard J-F, Lefort V, Anisimova M, Hordijk W, Gascuel O. New Algorithms and Methods to Estimate Maximum-Likelihood Phylogenies: Assessing the Performance of PhyML 3.0. *Syst Biol* 2010; **59**: 307–21.

4 Rambaut A, Lam TT, Max Carvalho L, Pybus OG. Exploring the temporal structure of heterochronous sequences using TempEst (formerly Path-O-Gen). *Virus Evol* 2016; **2**. DOI:10.1093/ve/vew007.

5 Shapiro B, Rambaut A, Drummond AJ. Choosing Appropriate Substitution Models for the Phylogenetic Analysis of Protein-Coding Sequences. *Mol Biol Evol* 2006; **23**: 7–9.

6 Suchard MA, Lemey P, Baele G, Ayres DL, Drummond AJ, Rambaut A. Bayesian phylogenetic and phylodynamic data integration using BEAST 1.10. *Virus Evol* 2018; **4**: vey016.

7 Rambaut A, Drummond AJ, Xie D, Baele G, Suchard MA. Posterior summarisation in Bayesian phylogenetics using Tracer 1.7. *Syst Biol* 2018; published online April 27. DOI:10.1093/sysbio/syy032.

8 WHO. Situation Report: Zika virus, Microcephaly, Guillain-Barré syndrome. 20 January 2017. 2017 http://apps.who.int/iris/bitstream/handle/10665/253604/zikasitrep20Jan17-eng.pdf?sequence=1.

9 International Air Transport Association. Passenger Intelligence Services (PaxIS). Montreal, Canada, 2017 http://www.iata.org/services/statistics/intelligence/paxis/Pages/index.aspx.

10 PAHO. Digitized Zika cases and incidence rates by epidemiological week from PAHO, provided by Andersen lab. 2018 https://github.com/andersen-lab/zika-epidemiology/tree/master/paho_case_numbers.

11 Lourenço J, Monteiro M de L, Valdez T, Rodrigues JM, Pybus O, Faria NR. Epidemiology of the Zika Virus Outbreak in the Cabo Verde Islands, West Africa. *PLOS Curr Outbreaks* 2018; : pii: ecurrents.outbreaks.19433b1e4d007451c691f138e1e67e8c.

12 Monteiro M de LS, Fortes AR. Boletim Informativo Semanal - Infeção por Vírus Zika (ZIKV) Ano 2016 (Semanas 1 a 21): http://www.minsaude.gov.cv/index.php/documentosite/zika-1/341-boletim-

informative-semanal-da-infecao-por-virus-zika-semana-21-ano-2016/file. Santiago, Cabo Verde, 2016.

13 United Nations. 2017 Revision of World Population Prospects. 2017 https://population.un.org/wpp/.

14 Kraemer MU, Sinka ME, Duda KA, *et al.* The global distribution of the arbovirus vectors Aedes aegypti and Ae. albopictus. *eLife* 2015; **4**: e08347.

15 Sassetti M, Zé-Zé L, Franco J, *et al.* First case of confirmed congenital Zika syndrome in continental Africa. *Trans R Soc Trop Med Hyg* 2018; **112**: 458–62.
